# Potential impact of task-shifting on costs of antiretroviral therapy and physician supply in Uganda

**DOI:** 10.1186/1472-6963-9-192

**Published:** 2009-10-21

**Authors:** Joseph B Babigumira, Barbara Castelnuovo, Mohammed Lamorde, Andrew Kambugu, Andy Stergachis, Philippa Easterbrook, Louis P Garrison

**Affiliations:** 1 Pharmaceutical Outcomes Research and Policy Program, School of Pharmacy, University of Washington, Seattle, WA, USA; 2 Infectious Diseases Institute, Makerere University, Kampala, Uganda; 3 Departments of Epidemiology and Global Health, School of Public Health & Community Pharmacy, University of Washington, Seattle, WA, USA

## Abstract

**Background:**

Lower-income countries face severe health worker shortages. Recent evidence suggests that this problem can be mitigated by task-shifting--delegation of aspects of health care to less specialized health workers. We estimated the potential impact of task-shifting on costs of antiretroviral therapy (ART) and physician supply in Uganda. The study was performed at the Infectious Diseases Institute (IDI) clinic, a large urban HIV clinic.

**Methods:**

We built an aggregate cost-minimization model from societal and Ministry of Health (MOH) perspectives. We compared physician-intensive follow-up (PF), the standard of care, with two methods of task-shifting: nurse-intensive follow-up (NF) and pharmacy-worker intensive follow-up (PWF). We estimated personnel and patient time use using a time-motion survey. We obtained unit costs from IDI and the literature. We estimated physician personnel impact by calculating full time equivalent (FTE) physicians saved. We made national projections for Uganda.

**Results:**

Annual mean costs of follow-up per patient were $59.88 (societal) and $31.68 (medical) for PF, $44.58 (societal) and $24.58 (medical) for NF and $18.66 (societal) and $10.5 (medical) for PWF. Annual national societal ART follow-up expenditure was $5.92 million using PF, $4.41 million using NF and $1.85 million using PWF, potentially saving $1.51 million annually by using NF and $4.07 million annually by using PWF instead of PF. Annual national MOH expenditure was $3.14 million for PF, $2.43 million for NF and $1.04 for PWF, potentially saving $0.70 million by using NF and $2.10 million by using PWF instead of PF. Projected national physician personnel needs were 108 FTE doctors to implement PF and 18 FTE doctors to implement NF or PWF. Task-shifting from PF to NF or PWF would potentially save 90 FTE physicians, 4.1% of the national physician workforce or 0.3 FTE physicians per 100,000 population.

**Conclusion:**

Task-shifting results in substantial cost and physician personnel savings in ART follow-up in Uganda and can contribute to mitigating the heath worker crisis.

## Background

Lower-income countries face severe health worker shortages and increasing demand for healthcare including antiretroviral therapy (ART). As a solution to this problem, stakeholders have proposed task-shifting--delegation of specific aspects of health care to less specialized health workers. [1-5] Task-shifting has been shown to be effective in high-income countries where appropriately trained and supervised lower-level cadres perform delegated tasks as well or better than physicians. [6-8] Emerging evidence from randomized trials as well as observational studies suggests that task-shifting is effective in lower-income countries. A recent randomized controlled trial in South Africa found that nurses were non-inferior to doctors when monitoring the treatment of HIV patients on ART[9]. Another cluster randomized trial in Uganda found that patients receiving home-based support, monitoring and drug delivery by lay workers with 6-monthly routine evaluation achieved favorable and comparable outcomes--mortality and virologic failure--to patients receiving facility-based care with monthly visits for drug refill and 3-monthly evaluation[10]. Task-shifting to non-physician clinicians in Malawi[11,12] and to non-physician clinicians and nurses in Zambia[13] did not compromise quality of care. Task-shifting to nurses in antiretroviral therapy in South Africa resulted in comparable outcomes while greatly increasing access[14]. When care teams included community health workers in addition to regular health providers mortality and loss to follow-up reduced significantly[15]. Given its effectiveness, we estimated the potential impact of task-shifting on costs and physician personnel supply in Uganda.

## Methods

### Study design and setting

We built an aggregate cost-minimization model to assess the potential cost impact of task-shifting for ART follow-up in Uganda from the societal and ministry of health (MOH) perspectives. The societal perspective includes costs of patients' time, their transport and health worker costs while the MOH perspective includes only health worker costs. Cost-minimization studies assume that health outcomes between comparators are equivalent. We obtained model inputs from primary surveys and the published literature. We excluded treatment and overhead costs; they do not differ by follow-up method. We conducted the study at the Infectious Diseases Institute (IDI), Makerere University in Kampala, Uganda. The IDI is a regional center of excellence in HIV/AIDS treatment, prevention, training, and research in Africa and operates an out-patient HIV/AIDS clinic with more than 5,000 patients on ART.

### Patient follow-up and task-shifting

IDI implements monthly ART follow-up using three algorithms: 1) physician-intensive follow-up (PF), 2) nurse-intensive follow-up (NF), and 3) pharmacy worker-intensive follow-up (PWF). Follow-up is organized in 6-month time blocks. In PF, the standard of care, patients have a physician visit (triage--physician--pharmacy) every month. In NF, a form of task-shifting, patients have a nurse visit (triage--ART-trained nurse--pharmacy) from month 1 to 5 and a physician visit (triage--physician--pharmacy) at month 6. In PWF, another form of task-shifting, patients have a pharmacy refill visit (prescription refill only) at month 1 and 2, a nurse visit (triage--ART-trained nurse--pharmacy) at month 3, a pharmacy refill visit at month 4 and 5, and a physician visit (triage--physician--pharmacy) at month 6. Physician visits are built into NF and PWF so that physicians supervise lower-level cadres and monitor patient progress. This sequence of visits means that, annually, PF requires 12 triage visits, 12 physician visits, and 12 regular PW visits; NF requires 12 triage visits, 2 physician visits, 12 regular PW visits and 10 ART-trained nurse visits; and PWF requires 4 triage visits, 2 physician visits, 4 regular PW visits, 2 ART-trained nurse visits, and 8 refill PW visits. At IDI, sicker patients are more likely to get treatment through PF while patients who are doing well on treatment are more likely to be sent to NF and PWF.

### Estimation of health worker and patient time use

We performed a one-day time-motion survey on August 20, 2007 to estimate time use by health workers and patients. Survey details have been reported elsewhere[16]. Briefly, we identified records of each of 400 patients scheduled to attend the clinic and attached a structured questionnaire to track "time in" and "time out" for different health worker posts. Clinic personnel recorded "time in" when the patient arrived at their post and "time out" when they left. We used this information to estimate time use by health workers and time spent waiting by patients which were summed to obtain total time spent by patients.

### Estimation of costs

All costs are in US dollars. We obtained unit personnel costs (hourly wage) from IDI: $8.46 for physicians, $4.65 for nurses and $3.38 for pharmacy workers. We assumed the opportunity cost of patient time to equal per capita gross domestic product: Uganda's GDP per capita for 2007 was $1,900[17] which translates to a mean societal hourly wage of $0.99 assuming 48 forty-hour workweeks annually. This approach has been used previously[18]. We multiplied unit costs by time estimates to obtain personnel and patient costs per visit for physicians, nurses and pharmacy workers. These were multiplied by annual number of visits for each type of health worker and summed across different health workers (needed to implement a particular follow-up algorithm) to obtain total annual cost per patient per visit for PF, NF and PWF.

### National cost projections

We made national projections by multiplying annual cost per patient with number of persons in Uganda who need ART under two assumptions: 1) current ART access and 2) 100% ART access. The Uganda MOH estimates that up to 300,000 of the 1 million HIV-infected patients in the country need treatment with ART. ART access in Uganda is about 33% (9%-68%)[19] or 99,000 people on ART. The national projections assume that ART clinics in the country are similar to IDI.

### Physician personnel impact of task-shifting

We estimated the potential physician personnel impact of task-shifting in terms of full-time equivalent (FTE) physicians saved. We multiplied physician time per visit by annual number of physician visits required for PF, NF and PWF to obtain physician time per patient per year. We then multiplied time by patient load under either access assumption to obtain total physician hours required to implement PF, NF or PWF nationally. We divided this by number of direct patient contact hours per physician per year calculated by assuming 48 40-hour workweeks per year and an 80% direct patient contact by physicians (based on judgment by IDI clinicians). We compared the physician FTEs between follow-up algorithms and with the total number of physicians in Uganda--estimated by WHO to be 2,209[20].

### Sensitivity analysis

We performed univariate sensitivity analysis to examine impact of different parameters on cost and physician personnel impact. We used 95% confidence intervals as the low and high estimates when available. When we did not have confidence intervals, we used ± 50% for costs and ± 20% for other inputs.

## Results

### Time use

Table 1 shows per visit time use by health workers and patients (in hours) for the 400 participants in the time-motion survey. Mean time use by health workers ranged from 0.24 hours for nurses to 0.05 for refill PWs while waiting times ranged from 1.20 for physicians to 0 for refill PWs. Since means and medians are similar, impact of outliers was minimal.

**Table 1:** Personnel time use and patient waiting times for different types of health workers at the Infectious Diseases Institute clinic in Kampala, Uganda

	**Mean**	**SD**	**Median**	**Range**	**IQR**
**Physician**					
Personnel	0.14	0.09	0.12	0.05 - 0.58	0.08 - 0.17
Patient waiting	1.20	0.87	1.08	0.03 - 3.30	0.53 - 1.65
**Triage**					
Personnel	0.24	0.19	0.20	0.03 - 1.05	0.08 - 0.33
Patient waiting	0.33	0.30	0.24	0.03 - 1.67	0.18 - 0.38
**Nurse**					
Personnel	0.16	0.10	0.13	0.03 - 0.42	0.08 - 0.23
Patient waiting	0.08	0.08	0.05	0 - 0.28	0.02 - 0.12
**Regular PW**					
Personnel	0.10	0.10	0.08	0.02 - 0.65	0.05 - 0.12
Patient waiting	0.36	0.32	0.27	0.02 - 1.73	0.15 - 0.47
**Refill PW**					
Personnel	0.05	0.03	0.03	0.02 - 0.13	0.02 - 0.05
Patient waiting	-	-	-	-	-

SD--Standard Deviation, IQR--Inter Quartile Range, PW--Pharmacy Worker, HWU--Health Worker Utilization

### Costs

Table 2 shows unit costs, health worker and patient time use, and cost per patient per visit for different kinds of health workers which ranged from $2.51 for physicians to $0.22 for refill PWs. Table 3 shows annual cost per patient of follow-up from a societal perspective which were as follows: PF $59.88, NF $44.58 and PWF $18.66. Table 4 summarizes annual costs per patient of follow-up from a MOH perspective, which were as follows: PF $31.68, NF $27.28 and PWF $10.50.

**Table 2:** Per visit time use, unit costs and total costs of follow-up for different types of health workers at the Infectious Diseases Institute clinic, Kampala, Uganda

**Health worker type**	**Personnel time (hours)**	**Hourly wage ($)**	**Per visit cost of Personnel ($)**	**Waiting time (hours)**	**Total time lost (hours)**	OC of lost patient time ($)*	**Total per visit cost ($)**
Physician	0.14	8.46	1.18	1.20	1.34	1.33	2.51
Triage nurse	0.24	4.65	1.12	0.33	0.57	0.56	1.68
Nurse	0.16	4.65	0.74	0.08	0.24	0.24	0.98
Regular PW	0.10	3.38	0.34	0.36	0.46	0.46	0.80
Refill PW	0.05	3.38	0.17	0	0.05	0.05	0.22

* This is multiplied by the unit hourly wage for Ugandan's of $ 0.99, PW--Pharmacy Worker, OC--Opportunity Cost

**Table 3:** Per visit and annual costs of antiretroviral therapy follow-up for different types of health workers at the Infectious Diseases Institute clinic, Kampala, Uganda from a societal perspective

**Health worker type**	**Cost per visit ($)**	**Number of visits per year**			**Annual societal cost of follow-up ($)**		
		**PF**	**NF**	**PWF**	**PF**	**NF**	**PWF**

Physician	2.51	12	2	2	30.12	5.02	5.02
Triage	1.68	12	12	4	20.16	20.16	6.72
Nurse	0.98	0	10	2	0	9.80	1.96
Regular PW	0.80	12	12	4	9.60	9.60	3.20
Refill PW	0.22	0	0	8	0	0	1.76
Total	--	--	--	--	59.88	44.58	18.66

PW--Pharmacy Worker, PF--Physician Intensive Follow-up, NF--Nurse Intensive Follow-up, PWF--PW-intensive follow-up

**Table 4:** Per visit and annual costs of antiretroviral therapy follow-up for different types of health workers at the Infectious Diseases Institute clinic, Kampala, Uganda from a ministry of health perspective

**Health worker type**	**Cost per visit ($)**	**Number of visits per year**			**Annual payer cost of follow-up ($)**		
		**PF**	**NF**	**PWF**	**PF**	**NF**	**PWF**

Physician	1.18	12	2	2	14.16	2.36	2.36
Triage	1.12	12	12	4	13.44	13.44	4.48
Nurse	0.74	0	10	2	0	7.40	0.94
Regular PW	0.34	12	12	4	4.08	4.08	1.36
Refill PW	0.17	0	0	8	0	0	1.36
Total	--	--	--	--	31.68	27.28	10.5

PW--Pharmacy Worker, PF--Physician Intensive Follow-up, NF--Nurse Intensive Follow-up, PWF--PW-intensive follow-up

### National projections

At current ART access, from a societal perspective, Uganda is projected to spend $5.92 million using PF, $4.41 million using NF and $1.85 million using PWF potentially saving $1.51 million by using NF and $4.07 million by using PWF instead of PF. From a MOH perspective, the country would spend $3.14 million for PF, $2.43 million for NF and $1.04 for PWF, potentially saving $0.70 million by using NF and $2.10 million by using PWF instead of PF. At 100% ART access, from a societal perspective, Uganda is projected to spend $17.96 million using PF, $13.37 million using NF and $5.60 million using PWF, potentially saving $4.59 million for using NF and $12.37 million for using PWF instead of PF. From a MOH perspective, the country would spend 9.50 million for PF, 7.37 million for NF and $3.15 million for PWF, potentially saving $2.13 million by using NF $6.35 million by using PWF instead of PF.

### Personnel impact of task-shifting

At current ART access, Uganda needs 108 FTE doctors to implement PF and 18 FTE doctors to implement NF or PWF (both require 2 physician visits per patient per year). Using NF or PWF instead of PF would potentially free up 90 FTE physicians, 4.1% of the national physician workforce or 0.3 FTE physicians per 100,000 population. At universal access, Uganda needs 328 FTE doctors to implement PF and 55 FTE doctors to implement NF or PWF. Using NF or PWF instead of PF would potentially free up 273 FTE physicians, 12.4% of the national physician workforce or 0.9 FTE physicians per 100,000 population.

### Sensitivity analysis

NF costs per visit rose above PF costs per visit when nurse wages exceeded $7.40 per hour. Savings from task-shifting using NF exceeded savings from using PWF when pharmacy worker wages exceeded $4.20 per hour. PF costs per visit fell below NF costs per visit if physician wages fell below $5.30 per hour. National costs of implementing PF fell below national costs for implementing NF when time spent with physicians fell below 0.09 hours per visit. Results were robust to variations in all other parameters.

## Discussion

Task-shifting from physicians to nurses and pharmacy workers in ART follow-up can save Uganda $0.5 to $11.0 million and free up 4.1 to 14.8% of the nationally available physician FTEs annually. Under the same methods and assumptions, task-shifting can save Sub-Saharan Africa--with 9.7 million patients in need of ART (31% access)[21] and 150,714 practicing physicians[20]--$0.476 to $2.769 billion and free up 4.1 to 5.9% of the physician FTEs available on the continent. To the best of our knowledge, this is the first study to estimate the cost and personnel impact of task-shifting in Uganda and other lower-income countries.

We used a cost-minimization model and did not measure quality of care, patient satisfaction, morbidity, mortality and adherence. Although data from IDI indicate that short-term outcomes--CD4 cell count, incidence of opportunistic infections and self-reported adherence--do not differ between patients attending regular care and those attending the pharmacy refill program,[22] outcomes may have been different for PF, NF and PWF.

Studies like ours of the comparative effectiveness of task-shifting are prone to selection bias: patients with a favorable prognosis are likely to be seen by lower level cadres as is the case at the IDI clinic where our study was performed. To minimize this bias, randomized studies of the effectiveness and cost-effectiveness of task-shifting are required. Two such trials have reported that task-shifting is effective and results in outcomes that are similar to care with doctors[9,10]. A review of on-line trial registers revealed that another trial to evaluate the effectiveness and cost-effectiveness of nurse-led versus doctor-led ART is currently ongoing in the Free State in South Africa.

The time-motion survey was performed in one day as an evaluation of patient flow at IDI. Although results were robust to time variations in sensitivity analysis, they may not be generalizable to other days. The IDI clinic is not generalizable to other clinics in Uganda because it is better staffed and better funded than most government clinics.

We recognize that task-shifting is not without challenges. Lower cadres to whom tasks are shifted must be trained to replace physicians; our estimates of physician savings from task-shifting would need a commensurate increase in the supply of nurses and PWs. And without appropriate training on HIV management, tools and supervision, they may overlook other potentially serious conditions such as tuberculosis which physicians may have been able to recognize. Task-shifting may lead to a higher rate of referral to physicians and increased use of laboratory and radiological tests with associated costs. Task-shifting may take lower-level cadres from their regular job. Nurses and PWs, faced with additional responsibilities and competencies, may demand greater remuneration although this is offset by limited (almost non-existent) foreign demand that precludes their out migration. Moreover, they cannot secure wages as high as those received by physicians. Future studies might explore the impact of these on cost and personnel savings.

The Global Health Workforce Alliance is proceeding in two strategic directions: accelerating action in countries and addressing global constraints that impede country-level action[23]. We view task-shifting as an economically attractive direction for accelerating action in countries with severe shortages despite political, financial and implementation constraints. This is consistent with the key recommendations of the Kampala declaration of the Global Health Workforce Alliance[24]. Between 2000 and 2005, Uganda trained 650 physicians at a cost of $11,600 each compared to 6,585 nurses at a cost of $730 each (government-subsidized) and $2,500 each (privately sponsored)[25]. Therefore countries can increase nurse and pharmacy worker numbers in a relatively short time. Still we recognize, as was eloquently discussed by Philips et al,[1] that task-shifting is not a panacea for the workforce crisis but part of an overall strategy to increase, retain, and sustain health workers.

## Conclusion

Task-shifting from physicians to nurses and pharmacy workers in ART follow-up has the potential to save money and physician personnel in lower-income settings such as Uganda. If cost offsets are used efficiently and physician FTEs saved are deployed usefully, task-shifting can mitigate the current health worker shortage and improve the provision of ART and other healthcare services in this setting.

## Competing interests

All authors declare that they have no competing interests. We received no funding for this study.

## Authors' contributions

JBB conceived of the study and participated in designing the study, collecting data, performing the cost analysis and drafting the manuscript. BC participated in designing the study, collecting data and drafting the manuscript. ML participated in designing the study, collecting data and revising the manuscript. AK participated in designing the study, collecting data and revising the manuscript. AS participated in designing the study and revising the manuscript. PE participated in designing the study, collecting data and revising the manuscript. LPG participated in designing the study, performing the cost analysis and revising the manuscript. All authors read and approved the final manuscript.

## Pre-publication history

The pre-publication history for this paper can be accessed here:


